# A Novel Approach to Teaching the Cervical Exam: A Versatile, Low-Cost Simulation for Labor and Delivery Learners

**DOI:** 10.7759/cureus.20235

**Published:** 2021-12-07

**Authors:** Jamie D Perry, Jill M Maples, Heather N Deisher, Hayley Trimble, Jaclyn V van Nes, Kaitlin Morton, Nikki B Zite

**Affiliations:** 1 Department of Obstetrics and Gynecology, University of Tennessee Graduate School of Medicine, Knoxville, USA; 2 Brookwood Women’s Health PC, Brookwood Baptist Medical Center, Birmingham, USA; 3 Obstetrics and Gynecology, Pikeville Medical Center, Pikeville, USA; 4 Kinesiology, Recreation, and Sport, University of Tennessee, Knoxville, USA

**Keywords:** fetal scalp electrode placement, artificial rupture of membranes, simulation model, cervix, effacement and dilation, simulation, obstetrics, labor and delivery

## Abstract

This technical report describes the making of cervical exam models that can be used to teach cervical dilation and effacement, with the versatility to teach additional obstetrical skills including artificial rupture of membranes (AROM) and fetal scalp electrode (FSE) placement. These models, primarily constructed from materials that are low cost and/or easily accessible within a healthcare setting, can be used to educate nurses, medical students, residents, and other healthcare professionals to improve the evaluation of the labor progress.

## Introduction

A critical skill for providers working with obstetric patients is the ability to perform an efficient and accurate cervical exam, which is considered a cornerstone in the management of labor [1]. Historically, after being introduced to cervical exams on a plastic model, new learners are taught to measure cervical dilation (opening) and effacement (thinning) by examining a cervix along with an experienced provider, and then comparing exams [2]. This classic training method results in increased examinations, which can cause discomfort and increase the risk of infection, especially after amniotic membranes are ruptured. Because of this, simulating cervical examinations for training is an attractive option.

Medical simulation in Obstetrics and Gynecology has gained popularity and has proven useful in teaching a variety of obstetrical skills, including cervical examinations [3,4]. Several commercial models to teach cervical examinations have been previously described [1,2,5-9] and vary widely in terms of cost and fidelity. Typically, the most accessible, low-cost models are lower in fidelity, including plastic dilation charts, cervical stackable models, and paper models. For example, plastic board models commonly used in healthcare settings typically have raised circles ranging in diameter from 1 to 10 centimeters, which represent the various stages of cervical dilation during labor. The learner both visualizes and palpates the circles in order to learn these stages. While the plastic board model is acceptable for teaching cervical dilation, it is ineffective in teaching effacement. It is also an unrealistic model for a real cervix and has no versatility in terms of teaching other obstetric technical skills.

High-fidelity cervical task trainers such as the Life/Form Cervical Dilation and Effacement models (Nasco, USA) are commercially available, but are expensive and, therefore, less accessible. These are silicone-based, 3D models designed to teach the learner cervical dilation with both visual and tactile sensations. This type of model also has set effacements associated with various cervical dilations, thereby adding an additional component that basic models lack. While these models teach cervical exams, they often lack the adaptability to teach other important obstetric skills.

Only a few other publications describe the creation of handmade models suitable for teaching cervical exams [8, 9]. The model described by Shea and Rovera [8] is a low-cost model made from citrus fruit and tube socks. While this model is excellent in terms of simulating cervical dilation and effacement, it utilizes perishable components which degrade over time and need to be replaced. The model described by Pratinidhi et al. [9] consists of a rotating drum with rubber-lined holes of various sizes mounted to a shaft so that it can rotate within a wooden box. This model teaches cervical dilation with visual sensations and palpation but does not teach effacement.

Given the limitations of the previously discussed cervical exam models, we sought to create an affordable and reproducible cervical dilation and effacement model with the versatility to teach other obstetric technical skills, such as artificial rupture of membrane (AROM) and fetal scalp electrode (FSE) placement. Therefore, the purpose of this technical report is to describe a novel and versatile cervical dilation and effacement model. This model was first described in a poster presented at the 2016 Council on Resident Education in Obstetrics and Gynecology (CREOG) and the Association of Professors of Gynecology and Obstetrics (APGO) Annual Meeting in New Orleans, LA on March 3, 2016.

## Technical report

Construction of the cervical model

**Figure 1:**
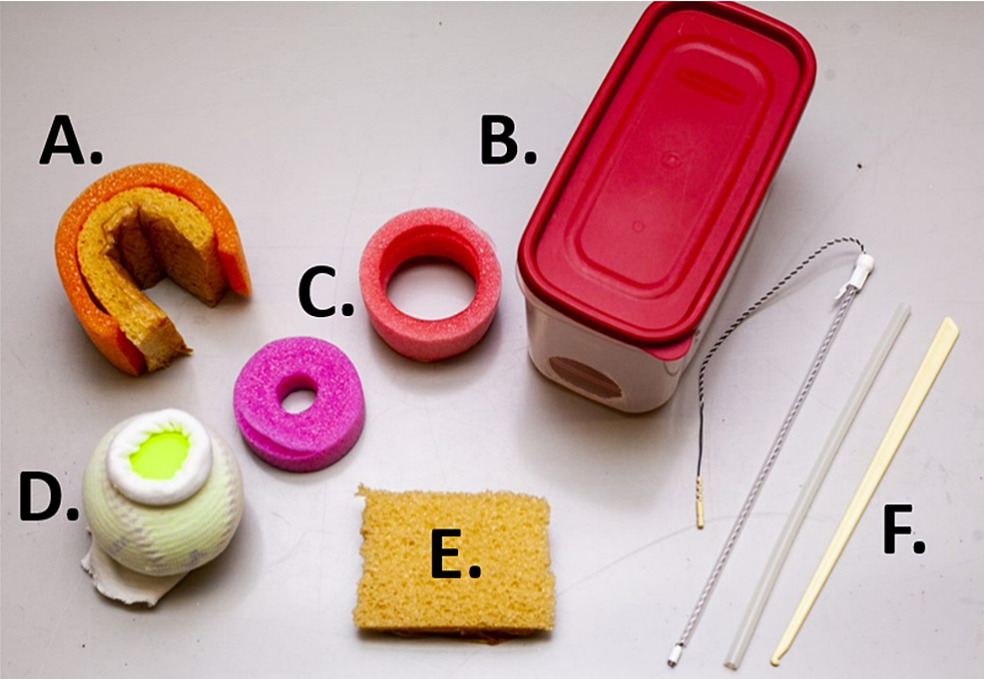
Various components used to create a novel, cervical exam model. The following items were used to create a novel, cervical exam model: A. Ioban-covered foam in cut pool noodle to simulate vaginal walls; B. plastic container with hole cut on end; C. cut pool noodles of various size; D. compression stockinette rolled over softball; E. second, smaller piece of foam covered in Ioban; F. fetal scalp electrode and plastic amniotomy hook.

A model to teach cervical dilation and effacement was created using a standard-sized softball, orthopedic compression stockinette (or tubular bandage), a foam pool noodle, a rigid container, and other materials chosen for their low cost and/or easy accessibility within a healthcare setting (Figure 1). The items chosen to create this model are easily interchangeable to allow for the model to be adapted to teach obstetrical skills in addition to cervical exams.

The materials/supplies and tools needed to build and adapt the model are listed below.

Materials and Supplies

Five-10 12-inch (30.5 cm), standard-sized softballs

One roll of orthopedic compression stockinette, tubular bandage, or similar product (5-10 cm flat width × at least 250 cm in length)

One foam pool noodle

One sheet of 3M Ioban drape (3M, Maplewood, MN, USA)

2-cm thick foam (memory or upholstery) sheet

24×11×13 cm plastic food storage container

Gloves

Water-soluble lubricant

Water balloons (approximately 5 cm in length, with the capacity to be filled so that the filled circumference measures approximately 30 cm)-Optional (if practicing AROM)

Absorbent material (to clean spills from AROM simulation)-Optional (if practicing AROM)

Tools

Scissors

Utility or craft knife (or other sharp cutting tool, such as a scalpel)

Centimeter tape measure to set cervical diameters

Sandpaper

Plastic amniotic membrane perforator (e.g. amniotomy hook)-Optional (if practicing AROM)

Fetal scalp electrode-Optional (if practicing FSE placement)

Model assembly

Cut the compression stockinette to approximately 25 cm length.

**Figure 2:**
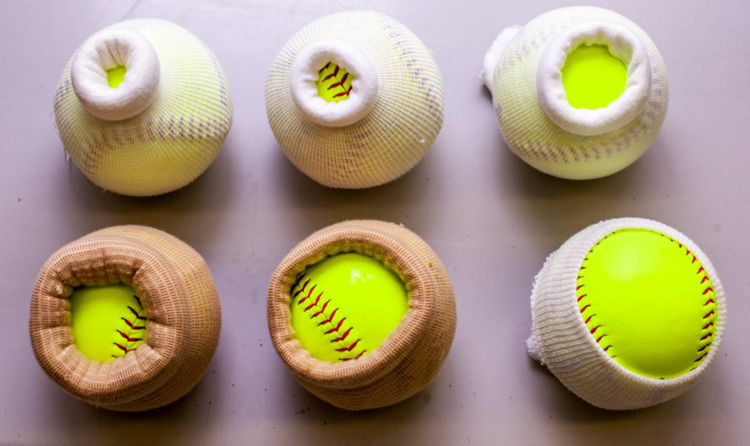
Various dilation and effacement combinations. Models shown range in size from 1 to 10 cm dilation with varying degrees of effacement.

Place softball in one end of stockinette and roll the other side until desired diameter and effacement are achieved (Figure 2).

Using a knife (or other sharp cutting tool, such as a scalpel), cut an 8 cm length of pool noodle, and then, cut along the vertical axis to create an arch.

Use scissors to cut a 16×8 cm and a 9×9 cm piece of foam and cover in Ioban drape. Set the smaller (9×9 cm) piece to the side for now, this will serve as the posterior vaginal wall.

**Figure 3:**
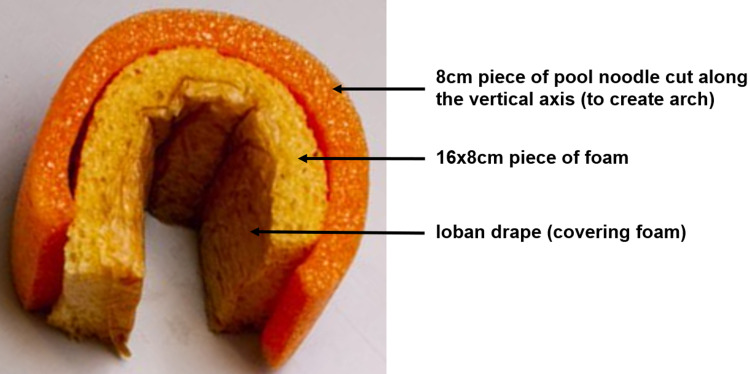
Assembled components of vaginal arch and walls.

Place the larger (16×8 cm) piece of foam inside the 8 cm pool noodle arch to create the anterior and lateral vaginal walls (Figure 3).

**Figure 4:**
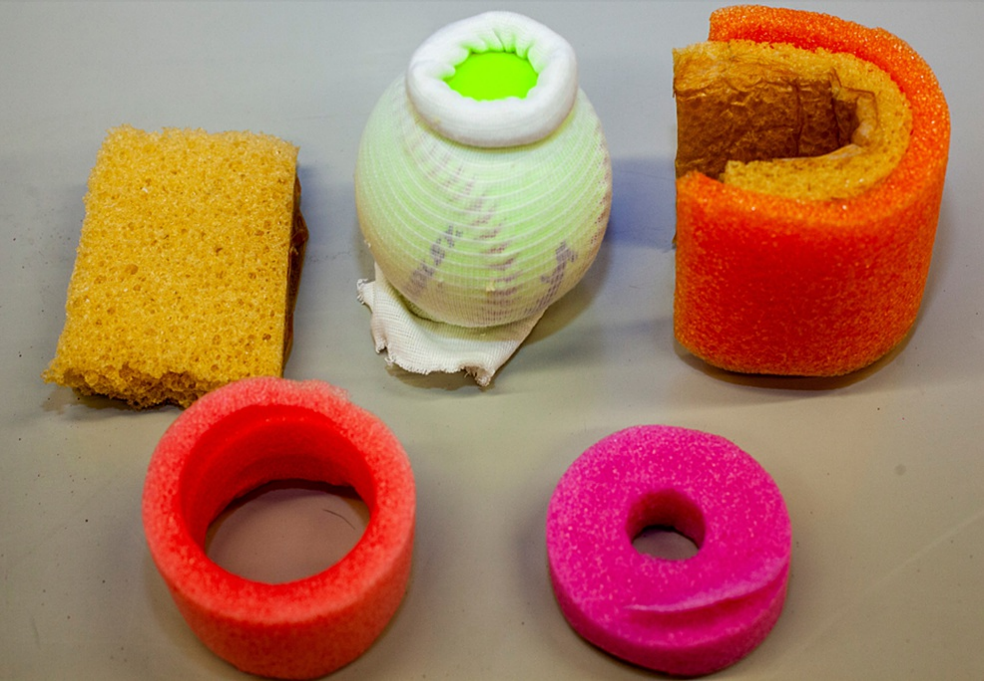
Components of the model that will be placed in plastic container. Shown in the top row, from left to right, is the smaller 9×9 cm foam piece covered in Ioban, the softball in the compression stockinette, and the assembled foam and pool noodle (forming the vaginal arch/walls). Shown in the bottom row are additional pieces of pool noodle, which will serve to stabilize the softball when placed in the plastic container.

The components shown in Figure 4 will be placed inside the plastic container. These include the smaller 9×9 cm foam piece covered in Ioban, the softball in the compression stockinette, and the assembled foam and pool noodle (forms the vaginal arch/walls). Additional short pieces of pool noodle can be used to provide support behind the softball, preventing movement during use.

**Figure 5:**
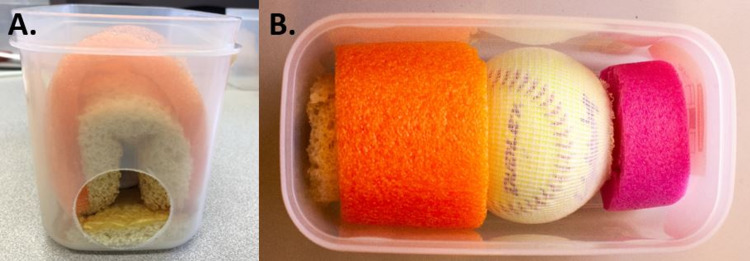
Placement of components inside the plastic container. A. (frontal view) and B. (superior view) showing the components placed inside the plastic container.

Use knife or sharp tool to create a circular opening in one end of the plastic container, approximately 5 cm in diameter (see Figures 1 and 5A). Sand the edges of the hole until smooth to avoid learner discomfort. Place the smaller 9×9 cm foam piece in the plastic container on the end with the 5 cm hole, Ioban-covered side up. Next, place the assembled foam and pool noodle vaginal arch directly on top of the smaller 9×9 cm piece of foam (Figure 5A).

Place the softball (already in the compression stockinette and adjusted to the desired dilation/effacement) directly behind the vaginal arch (Figure 5B).

Secure the softball by placing additional piece(s) of pool noodle behind the softball (Figure 5B).

Place the lid on the box to create a blind examination.

If multiple boxes are used, the back of the box can be labeled with the correct dilation and effacement. Alternatively, if a limited number of boxes are used, the dilation and effacement can be written on the back of each softball, which can be quickly exchanged.

**Figure 6:**
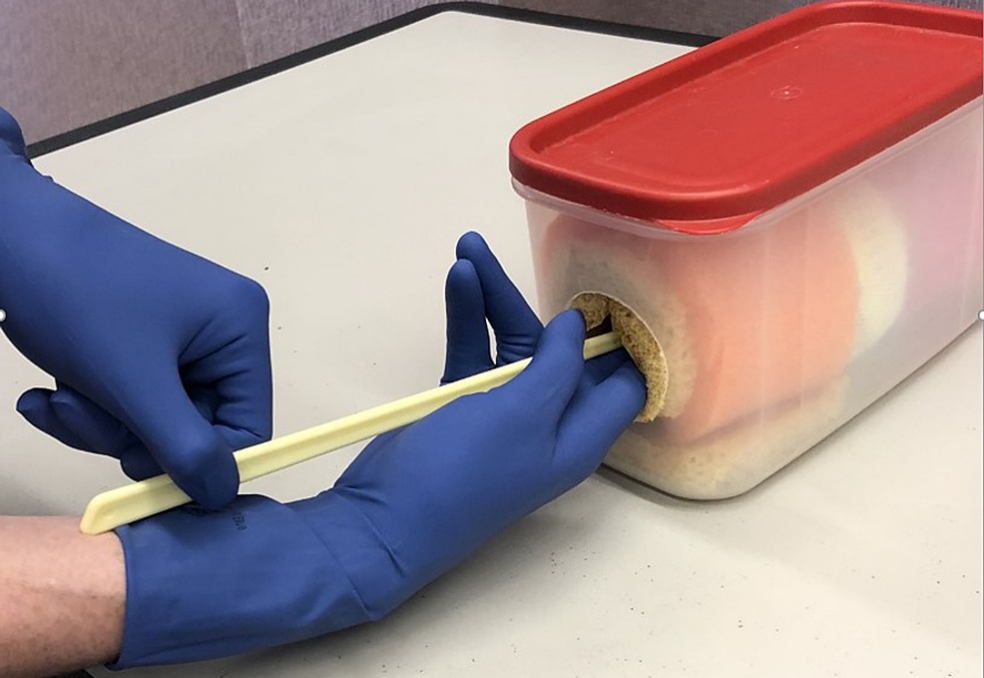
Learner practicing amniotic membrane rupture with amniotomy hook and water-filled balloon in place of softball.

The assembled models can be used to practice additional obstetrical skills, such as FSE placement or AROM (Figure 6). To practice FSE placement, the FSE would be inserted into the softball. To practice AROM, a water-filled balloon would be set in the model, in place of the softball, and absorbent padding (or a towel) would be placed under the model. A standard water balloon, approximately 5 cm in length, can be filled with approximately 500 ml of water to achieve a circumference similar to that of the softball (30 cm). The amount of fluid released during AROM is variable, ranging from 0 to 500 ml. Practicing AROM with the water-filled balloon allows the learner to become familiar with the practice of orienting the instrument and applying pressure against the balloon (representing the amniotic sac) with only blind palpation and simulates a realistic fluid release. This is not an insignificant amount of liquid, and it is recommended to have extra absorbent towels or other similar material to clean spilled water in between simulations. Additional absorbent material inside the box is also recommended and this simulation could be performed without the stockinette.

## Discussion

Several studies have investigated the benefit of using simulation to teach obstetric skills, both for the purpose of increasing learner skills and confidence while reducing harm to patients [10]. A study by Nitsche et al. estimated that a new learner requires approximately 100 repetitions of a simulated cervical exam to achieve competence [2]. Additionally, they found that the accuracy of learner assessment decreased with higher cervical dilations and lower effacements [11]. This demonstrates the need for a durable model with the versatility to teach both components of the laboring cervical exam.

To date, this model has been used at a single, academic medical institution to teach resident physicians, medical students, and nursing students. To confirm validity of this model, future studies are needed. Ideally, a study would assess the skills of learners that have trained using this novel cervical exam model. As with previous models, a limitation of this model is the inability to use it to teach fetal station or position, but one could imagine a future model replacing the plastic container with a pelvic bone model and the softball with a model fetal head complete with sutures.

## Conclusions

We sought to create a low-cost, reproducible, and versatile model that can accurately teach a new learner cervical dilation, effacement and have the ability to practice additional obstetrical skills. The novelty of this model is primarily in its ability to teach not only dilation but also cervical effacement while offering a more realistic, blinded exam. Additionally, it provides versatility, allowing for instruction on both FSE placement and amniotic membrane rupture. Another strength of the model is that it is easily reproducible and inexpensive. It costs approximately US$15 to produce one of these models. The models can be completely assembled in approximately 25 minutes and stored for future use. We recommend having several boxes and leaving the models assembled, but if a smaller budget is available, having one box and switching out the softball is reasonable.

In conclusion, this technical report describes a novel model of cervical examination using a softball and compression stockinette in a rigid container. This model has clear advantages over previous models including its cost-effectiveness, durability, easy reproducibility, and versatility. In place of other models, this model can be used to educate nurses, medical students, residents, and other healthcare professionals with the ultimate goal of improving the evaluation of labor progress.
